# Repeated Intrastriatal Botulinum Neurotoxin-A Injection in Hemiparkinsonian Rats Increased the Beneficial Effect on Rotational Behavior

**DOI:** 10.3390/toxins10090368

**Published:** 2018-09-11

**Authors:** Alexander Hawlitschka, Carsten Holzmann, Andreas Wree, Veronica Antipova

**Affiliations:** 1 Institute of Anatomy, Rostock University Medical Center, D-18057 Rostock, Germany; alexander.hawlitschka@med.uni-rostock.de (A.H.); veronica.antipova@medunigraz.at (V.A.); 2 Institute of Medical Genetics, Rostock University Medical Center, D-18057 Rostock, Germany; carsten.holzmann@med.uni-rostock.de; 3 Gottfried Schatz Research Center for Cell Signaling, Metabolism and Aging, Macroscopic and Clinical Anatomy, Medical University of Graz, A-8010 Graz, Austria

**Keywords:** botulinum neurotoxin-A, striatum, basal ganglia, donepezil, acetylcholine, 6-OHDA

## Abstract

Injection of botulinum neurotoxin-A (BoNT-A) into the striatum of hemiparkinsonian (hemi-PD) rats reduced apomorphine-induced rotation behavior significantly, for at least 3 months. Thereafter, rotation behavior increased again. We injected hemi-PD rats with 1 ng BoNT-A twice, the second injection following 6 months after the first one and tested the rats for apomorphine-induced rotations and spontaneous motor behaviors, i.e., corridor task and stepping test. To test the hypothesis that BoNT-A reduced striatal hypercholinism in hemi-PD rats, the acetylcholinesterase inhibitor donepezil was injected prior to separate apomorphine-induced rotation tests. In hemi-PD rats, the first BoNT-A injection led to a clear reduction of the apomorphine-induced rotations, and the second BoNT-A injection to a more massive and prolonged reaction. In hemi-PD rats whose apomorphine-induced rotation behavior was strongly reduced by an intrastriatal BoNT-A, subsequent donepezil injections led to significant increases of the rotation rate. Concerning corridor task and stepping test, neither first nor second BoNT-A injections changed hemi-PD rats’ behavior significantly. The data give evidence for the possibility of repeated intrastriatal administrations of BoNT-A, for treatment of motor symptoms in experimental hemi-PD over a longer time.

## 1. Introduction

**Figure 1:**
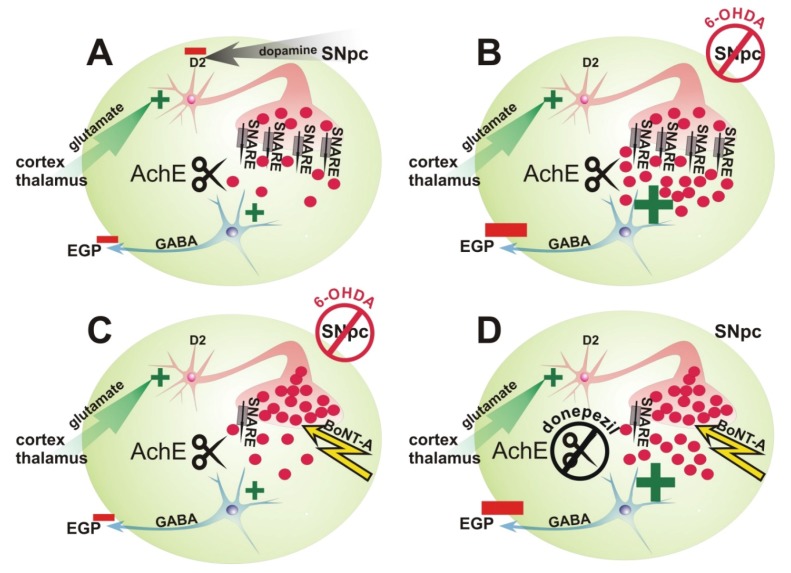
Theoretical concept of the ACh-dependency of the reduction of the apomorphine-induced rotation rate after unilateral BoNT-A treatment of hemiparkinsonian (hemi-PD) rats. (**A**–**D**) Scheme of the indirect pathway of the basal ganglia circuitry. Neurons, efferences and afferences of the striatum (green oval area) relevant for our experiments are shown. A large cholinergic interneuron is depicted with a stylized presynapse (red), the red circles representing ACh. The rectangles with an arrow (SNARE) symbolize the entirety of the *N*-ethylmaleimide-sensitive-factor attachment receptor complex, which conveys the fusion of transmitter vesicles with the presynaptic membrane. The scissors named with “AChE” represent acetylcholinesterase which cleaves ACh, and the blue neuron a medium sized spiny projection neuron (MSN), which inhibits the globus pallidus externus (EGP) neurons by GABA. (**A**) Under normal conditions tonically active cholinergic interneurons excite MSNs by release of ACh. These cholinergic interneurons are excited by glutamatergic input of motor, sensory, and prefrontal cortices and thalamus. Owing to their D_2_ receptors, cholinergic interneurons are inhibited by dopaminergic input from the substantia nigra pars compacta (SNpc). (**B**) After lesion of the SNpc with 6-OHDA the dopaminergic input to the striatum, and consequently, the inhibition of cholinergic interneurons is reduced, leading to a striatal extracellular hypercholinism, symbolized by a higher number of red circles. Therefore, GABA-ergic projection neurons to the EGP become overactive. (**C**) The injection of BoNT-A directly into the striatum cleaves the SNAP-25, an essential component of the SNARE complex in cholinergic presynaptic boutons. In consequence, the extracellular amount of ACh and the inhibition of the EGP should normalize. (**D**) After administration of the blood-brain barrier passing acethylcholinesterase inhibitor donepezil, the BoNT-A-induced reduction of extracellular ACh in hemi-PD rat striatum should be at least partially reversed, and the extracellular ACh concentration in the striatum increased again.

Parkinson’s disease (PD) is the most frequent neurodegenerative movement disorder, which mainly affects movement ability. PD is associated with a loss of dopaminergic neurons in the substantia nigra pars compacta (SNpc), leading to a lack of dopaminergic inhibition of tonically active cholinergic interneurons in the striatum (CPu) (Figure 1A,B). This causes a hypercholinism in the CPu, which is thought to contribute to the majority of motor symptoms in PD [1,2,3,4,5] (Figure 1B). Classic anticholinergic treatments ameliorate motor symptoms of PD, but acting systemically they also entail numerous unwanted side effects [6,7,8,9].

Botulinum neurotoxin-A (BoNT-A) cleaves synaptosomal-associated protein-25 (SNAP-25), a component of the vesicle fusion apparatus of the cholinergic presynaptic membrane. Therefore, it inhibits the release of acetylcholine (ACh) in the peripheral nervous system [10,11], and as we hypothesized in the central nervous system as well [12] (Figure 1C). Intrastriatal injection of 1 ng BoNT-A in hemiparkinsonian (hemi-PD) rats reverses apomorphine-induced rotations for at least 3 months [12,13,14,15,16,17]. During a time frame of 12 months after BoNT-A treatment, hemi-PD rats showed a gradual recurrence of the apomorphine-induced rotation rate [12,13,14].

**Figure 2:**
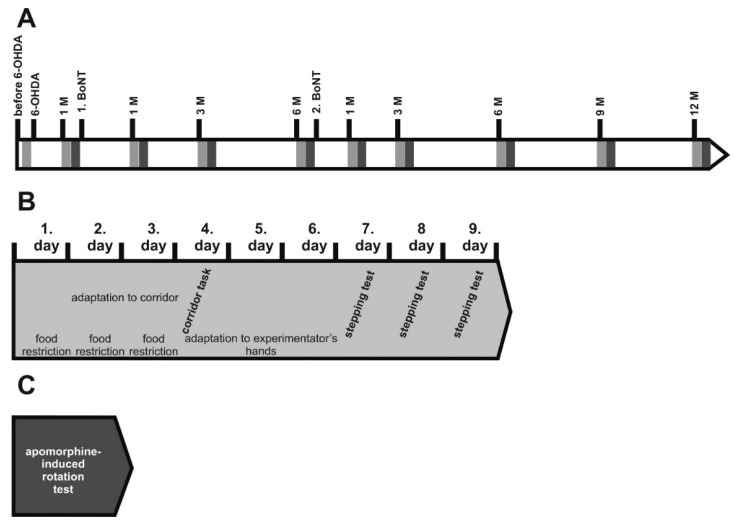
(**A**) Time points of 6-OHDA lesion and behavioral tests in rats after first (1. BoNT) and second (2. BoNT) BoNT-A or sham injection. Light grey rectangles symbolize batteries of corridor task and stepping test, dark grey rectangles apomorphine-induced rotation test. (**B**) The spontaneous behavior tests were performed as follows: Each series lasted 9 days. During the first 3 days, rats were food restricted and then 2 days adapted to the corridor task apparatus for 10 min each. Next day, the final corridor task was performed for 5 min. At the following 3 days, rats were handled by the experimenter for 5 min each day, and on the following 3 days, rats underwent the stepping test twice a day. (**C**) Finally, apomorphine-induced rotation test was performed for 40 min.

In this study, two aspects of hemi-PD and BoNT treatment were evaluated. First, we tested whether repeated intrastriatal BoNT-A injections were able to improve motor behavior in hemi-PD rats for a longer time period, resembling used clinical practice for BoNT-A treatments [18,19]. Thus, hemi-PD rats were intrastriatally injected with 1 ng BoNT-A 1 month and 7 months following 6-hydroxydopamine (6-OHDA) lesion (Figure 2), and underwent the apomorphine-induced rotation test, as well as the stepping test and corridor task for spontaneous motor behaviors.

In the second set of experiments, the effect of donepezil on apomorphine-induced rotation behavior in hemi-PD rats was investigated. Doing so, we wanted to test whether the decrease of the apomorphine-induced rotation rate after intrastriatal BoNT-A injection in hemi-PD rats was due to a reduction of extracellular ACh content in the CPu. We supposed that the observed decrease in the apomorphine-induced turning rate in BoNT-A-injected hemi-PD rats, was attributed to a reduction of ACh content in the treated CPu. However, direct evidence for a decrease of the extracellular ACh concentration in the CPu in hemi-PD rats after BoNT-A application, and a correlation between the hypothesized ACh decrease and the reduction of the apomorphine-induced turning rate, was not provided yet.

Assuming the observed beneficial effect of BoNT-A was a result of a reduction of striatal extracellular ACh content, an increase of the extracellular ACh should abrogate this effect. Experimentally, such an increase of ACh in the synaptic clefts of the CPu was provoked by application of the blood-brain barrier passing acetylcholinesterase (AChE) inhibitor donepezil (Figure 1D) [20]. Hemi-PD BoNT-A-treated rats underwent the apomorphine-induced rotation test up to 9 months after BoNT-A, and each again 72 h later following injection of donepezil 1 or 24 h ahead. Rotations of both tests were compared to separate the donepezil effect.

There is consistent information about the pharmacological half-life of donepezil in man, but not in rats. In the product information of donepezil-based medications (Aricept^®^, Full Prescribing Information; 2016 http://www.aricept.com/prescribing-and-patient-info), as well as in several publications [20,21,22,23,24,25], the half-life (t_1/2_ ) is denoted at about 70 h in man. However, in rats, divergent half-lifes were reported either in the range of 2 up to 3.5 h [26,27,28] or about 24 h [29]. To circumvent the divergent information about the t_1/2_ of donepezil, we studied experimental groups which were administered donepezil or sham donepezil 24 h or 1 h prior to the second rotation test at each monitoring point (Figures 7 and 9).

## 2. Results

### 2.1. Repetitive Intrastriatal BoNT-A Injection in Hemi-PD Rats

#### 2.1.1. Body Weight

**Figure 3:**
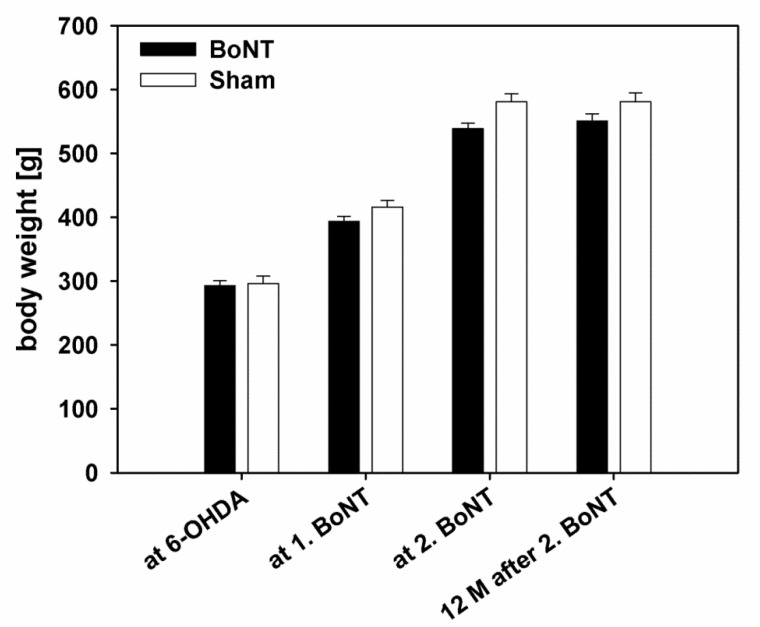
Body weights of both groups evaluated at the following time points: day of 6-OHDA injection, day of first (1. BoNT) and second (2. BoNT) BoNT-A or vehicle injections, as well as 12 months after the second BoNT-A or vehicle applications. Repetitive BoNT-A injection did not alter body weights, compared with vehicle injection in hemi-PD rats. All data are represented as mean ± SEM.

Both intrastriatal applications of BoNT-A were tolerated well by the animals and did not result in health problems. Thus, the development of body weight in hemi-PD rats was neither significantly altered by the first nor second BoNT-A injections, compared with sham-injected controls (F_1, 20_ = 1.091, *p* = 0.31). (Figure 3).

#### 2.1.2. Apomorphine-Induced Rotation Test

**Figure 4:**
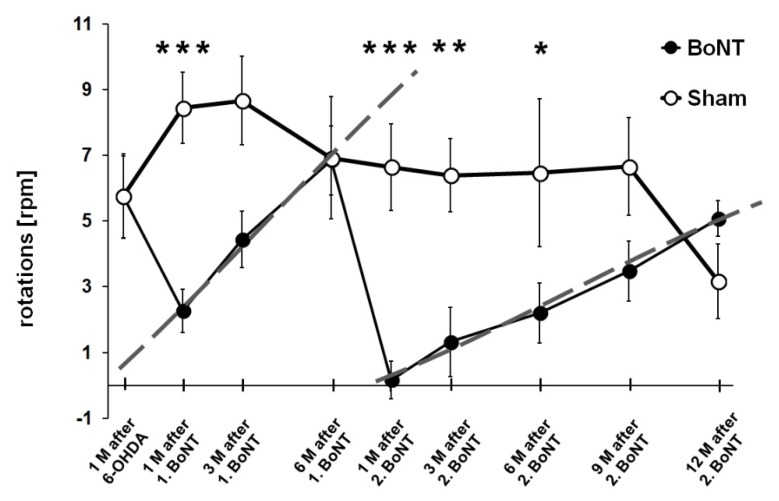
Apomorphine-induced rotation rates of hemi-PD rats repetitively treated with intrastriatal BoNT-A (*n* = 11, black dots) or vehicle (*n* = 6, light dots) 1 month and 7 months after 6-OHDA lesion. Vehicle solution has no significant effect on apomorphine-induced rotation behavior in hemi-PD rats. First BoNT-A reduces the turning rate significantly and temporally, the effect diminishing after 6 months. The effect of the 2. BoNT-A injection is more distinct and lasts longer than the 1. BoNT. Dashed grey lines mark linear regressions of the respective time dependencies of the apomorphine-induced rotation rates after 1. and 2. BoNT-A. Asterisks indicate significant difference (* *p* < 0.05, ** *p* < 0.01, *** *p* < 0.001). Data are represented as mean ± SEM.

Hemi-PD rats intrastriatally treated with BoNT-A had less apomorphine-induced rotation, compared with sham-injected animals (F_1, 20_ = 4.385, *p* = 0.048). Hemi-PD rats 4 weeks after 6-OHDA, showed about 6 apomorphine-induced turns per minute. In the present study, hemi-PD rats 1 month after BoNT-A showed about 2 apomorphine-induced rotations per minute, and thereby significant lower values compared with sham-injected hemi-PD rats (*p* = 0.001). Thereafter, a progressive resurgence of the BoNT-A-induced reduction of the apomorphine-induced rotation rate occurred, reaching values identical to sham-injected rats at 6 months (Figure 4). However, 6 months after the second BoNT-A injection using the same coordinates as before, an even more pronounced and significant decline of the turning rate to about 2 was found, resembling the 1-month value of the first BoNT-A injection (Figure 4). To fit the rotation data by a regression line, the parameter rpm/day was calculated for every post BoNT-A test point after the first and second application, and both slopes (m) of the regressions were calculated. The time dependent slope of the apomorphine-induced rotation rates after the first BoNT-A injection was m = 0.0296 rpm d^−1^, which after the second BoNT-A injection was significantly flatter (m = 0.0136 rpm d^−1^) (t_20_ = 2.531, *p* = 0.02) (dashed lines in Figure 4).

#### 2.1.3. Spontaneous Motor Tests

##### Adjusting Steps

The stepping test was used to evaluate forelimb akinesia, which is most likely due to bradykinesia of the affected limb [30,31,32,33]. Adjusting steps were measured on the non-lesioned and lesioned sides, for each animal group (BoNT-A- and sham-treated hemi-PD rats), in the forehand and backhand directions.

**Figure 5:**
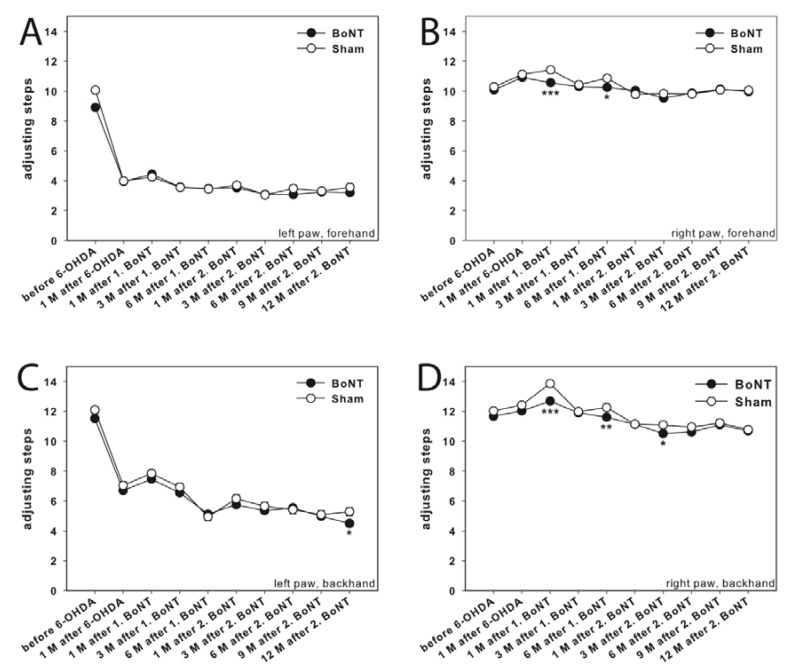
Stepping test in hemi-PD rats treated with repetitive intrastriatal BoNT-A or vehicle. In unlesioned rats, about 10–12 adjusting steps for the left (**A**,**C**) and right (**B**,**D**) forelimbs in forehand and backhand directions were counted. In hemi-PD rats, the use of the left forepaw was impaired in both the forehand (**A**) and backhand (**C**) directions. Neither 1. nor 2. BoNT-A nor sham injection changed the impairment of left and right forelimb steps in both the forehand (**A**,**C**) and backhand (**B**,**D**) directions in hemi-PD rats. Asterisks indicate significant differences compared to the sham group (* *p* < 0.05, ** *p* < 0.01, *** *p* < 0.001). Data are represented as mean ± SEM.

Before 6-OHDA lesion, no difference in the number of adjusting steps for the left and right forepaws, in forward and backward directions, was observed in either group (Figure 5A–D). All rats made about 9–12 steps in forward and in backward directions, with their left and right forepaws. 

Right side 6-OHDA lesion induced impairment in the performance of the left forelimb (contralateral to 6-OHDA, Figure 5A,C) during the adjusting step when the rats were moved forward and backward by the experimenter, the right forelimb (ipsilateral to 6-OHDA lesion) being unaffected (Figure 5B,D). No significant improvement or worsening of the left forepaw movements was monitored after the first, as well as after the second intrastriatal BoNT-A or sham injection (Figure 5A,C). At the same time, in 6-OHDA-lesioned rats, side stepping movements of the right forepaw in both forehand and backhand directions, were generally neither affected by ipsilateral BoNT-A nor sham injection up to 12 months (Figure 5B,D), with exception of single time points.

##### Corridor Task 

**Figure 6:**
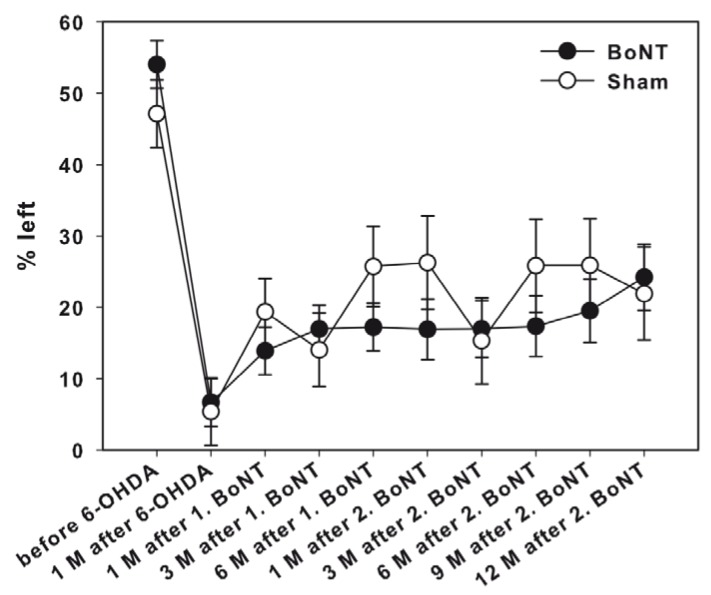
Corridor task in hemi-PD rats repetitively treated with intrastriatal BoNT-A or vehicle. Before 6-OHDA lesion, all animals equally retrieved pellets from either the left or right sides of the corridor apparatus. Right side hemi-PD rats significantly neglected the left corridor side. Neither 1. nor 2. BoNT-A nor sham injection improved contralateral sensorimotor integration in hemi-PD rats. Data are represented as mean ± SEM.

Two weeks before the 6-OHDA injection, rats were tested in the adjacent version of the corridor task. Preoperative screening showed that all animals performed an equivalent number of retrievals from each side of the corridor (about 50% of the total retrievals from either side) (Figure 6). Animals with right side hemi-PD exhibited a strong neglect of the left corridor side (contralateral to lesion): Only about 5% of retrievals were measured on the left side (Figure 6).

Neither the first ipsilateral intrastriatal BoNT-A nor sham injections in hemi-PD rats, improved a contralateral sensorimotor integration up to 6 months significantly (Figure 6). Moreover, the second intrastriatal injection of BoNT-A or vehicle demonstrated non-significant effects on sensorimotor (spatial) neglect for the side contralateral to the lesion during the next 12 months post-injection (Figure 6). 

### 2.2. Influence of Donepezil on Apomorphine-Induced Rotation Behavior 

In the second experiment, we investigated whether it could be the reduced extracellular ACh concentration that caused the reduction of the BoNT-A-related apomorphine-induced rotation rate in hemi-PD rats. We compared the results of two apomorphine-induced rotation tests performed with an interval of 72 h, whereby the first was performed without additional donepezil, and the second following injection of donepezil (2 mg kg^−1^ body weight (BW)), either 24 h or 1 h before apomorphine.

#### 2.2.1. Reaction of Rats to Donepezil 

Animals’ reaction to 2 mg kg^−1^ BW donepezil was qualitatively evaluated. Starting about 8–10 min after i.p. injection, rats showed donepezil-related gnawing. This donepezil-induced gnawing behavior lasted approximately for about 2.5 h. We also measured a decline of the body temperature of approximately 1 °C, 1 h after donepezil administration. Tremor, salivation, lacrimation, and increased defecation, indicative for threefold higher donepezil dosages used by others were not seen [23,27,34,35,36]. General motor activity seemed uninfluenced by donepezil. Moreover, no fasciculations mentioned after daily oral application of 2.5 mg kg^−1^ [37] were seen after a single dose applied i.p., as used in our study.

#### 2.2.2. Donepezil Injection 24 h Prior to the Second Rotation Test

**Figure 7:**
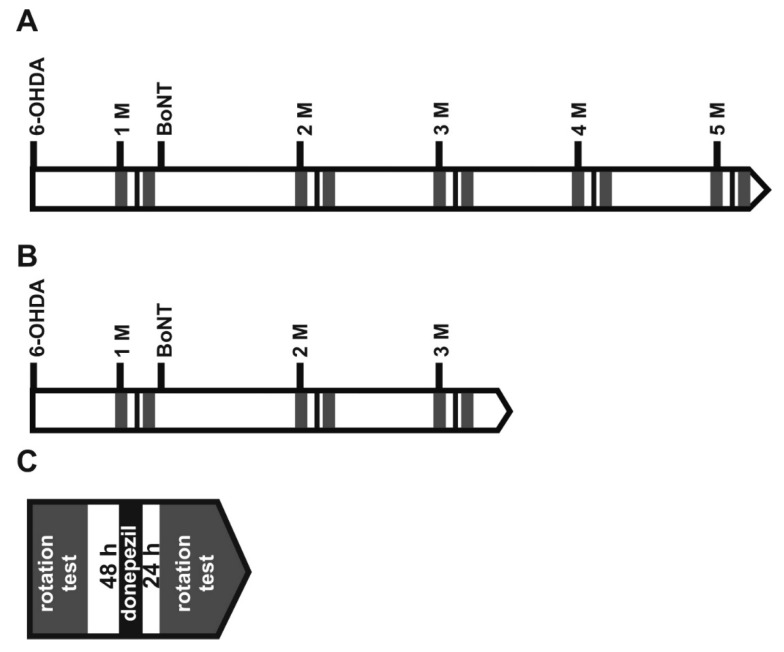
Time points of repetitive apomorphine-induced rotations (dark grey rectangles) in hemi-PD rats, before and after BoNT-A or sham injection, under the influence of donepezil (**A**) or sham donepezil (**B**). (**C**) The two apomorphine-induced rotations were performed as follows: Apomorphine rotations were tested 72 h apart. Furthermore, 24 h before the second rotation test, donepezil or vehicle was injected.

Two groups of hemi-PD rats were used. One group was intrastriatally injected with 1 ng BoNT-A (group 6-OHDA + BoNT-A), and the other with the solvent of BoNT-A (group 6-OHDA + sham BoNT-A). At 1, 2, 3, and 4 months after BoNT-A or vehicle application, the apomorphine-induced rotation rate was measured, and at each time point 48 h later, 2 mg kg^−1^ BW donepezil was injected i.p., and a further 24 h later, the second apomorphine-induced rotation test was performed (Figure 7).

**Figure 8:**
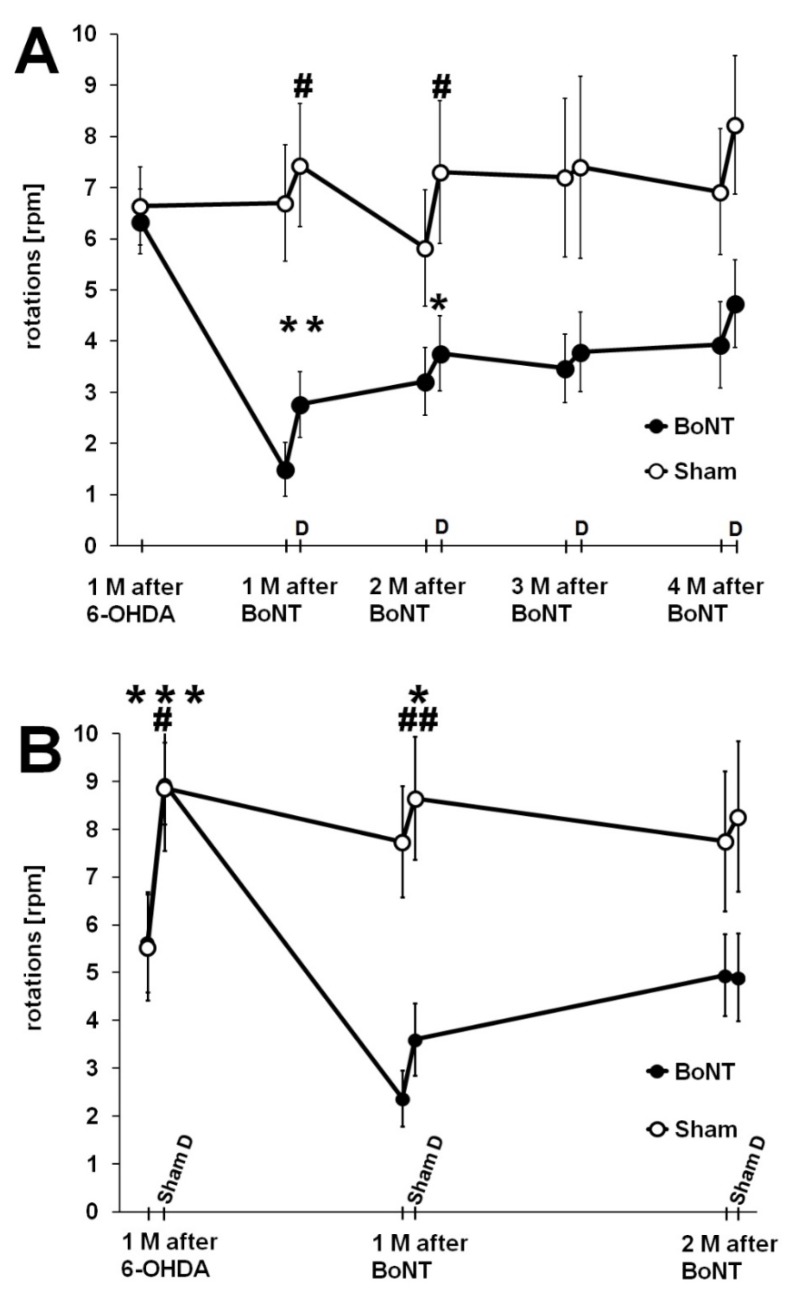
(**A**) Hemi-PD rats treated with intrastriatal BoNT-A (black dots) or vehicle (light dots) underwent apomorphine-induced rotation tests, before and 1 to 4 months after BoNT-A or sham BoNT-A. In the second apomorphine-induced rotation test under the influence of donepezil (2 mg kg^−1^ BW) injected 24 h ahead, the rotation rate significantly increased at time points 1 and 2 months after BoNT-A, as well as after sham BoNT-A. Asterisks mark significant donepezil-induced changes of the turning rate between the two associated rotations in the group which was treated with BoNT-A, hashes mark those in the group treated with sham BoNT-A. (**B**) Hemi-PD rats treated with intrastriatal BoNT-A (black dots) or vehicle (light dots) underwent apomorphine-induced rotation test, before and 1 to 2 months after BoNT-A or sham BoNT-A. In the second apomorphine-induced rotation test under the influence of sham donepezil injected 24 h ahead, rotation rates significantly increased at time points 1 month after 6-OHDA and 1 month after BoNT-A. Asterisks mark significant sham donepezil-induced changes of the turning rate between the two rotation tests of the BoNT-A-treated group, hashes mark significant changes in the group treated with sham BoNT-A. Asterisks and hashes indicate significant differences (* *p* < 0.05, ** *p* < 0.01, *** *p* < 0.001; ^#^ *p* < 0.05, ^##^ *p* < 0.01). Data are represented as mean ± SEM.

As expected, intrastriatal BoNT-A reduced apomorphine-induced rotations in hemi-PD rats (Figure 8A). Seventy-two h later the same rats having received donepezil 24 h before, showed significantly increased rotation rates (Figure 8A). 

However, a comparable donepezil effect in apomorphine-induced rotations was also found in sham BoNT-A-treated hemi-PD rats (Figure 8A). Significant differences between the two consecutive rotation tests—first without and second with donepezil—were found for the monitoring points at 1 and 2 months after the BoNT-A or sham BoNT-A administration (Figure 8A). 

#### 2.2.3. Sham Donepezil Injection 24 h Prior to the Second Rotation Test

Using a comparable experimental design, except for the donepezil injection prior to the second rotation, rats were injected with 0.9% NaCl solution 24 h prior to the second rotation test at the monitoring points 1 month after the 6-OHDA, and 1 and 3 months after BoNT-A injection in hemi-PD rats (Figure 8B). Remarkably, in these experiments we measured significant increases in the apomorphine-induced rotations 24 h after sham donepezil injection, 1 month after 6-OHDA lesion, as well as 1 month after BoNT-A injection or sham BoNT-A injection (Figure 8B).

#### 2.2.4. Donepezil Injection 1 h Prior to the Second Rotation Test

**Figure 9:**
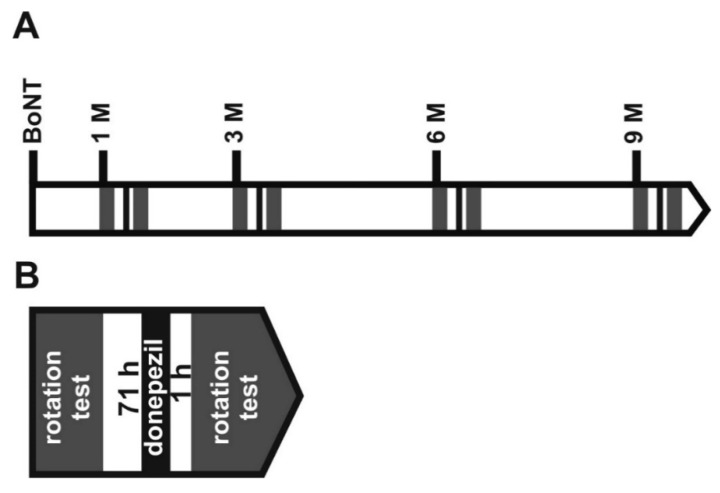
(**A**) Time points of repetitive apomorphine-induced rotations (dark grey rectangles) in hemi-PD rats, before and after BoNT-A or sham injection, under the influence of donepezil or sham donepezil. (**B**) The two apomorphine-induced rotations were performed as follows: Apomorphine rotations were tested 72 h apart. One h before the second rotation test, donepezil or vehicle was injected.

**Figure 10:**
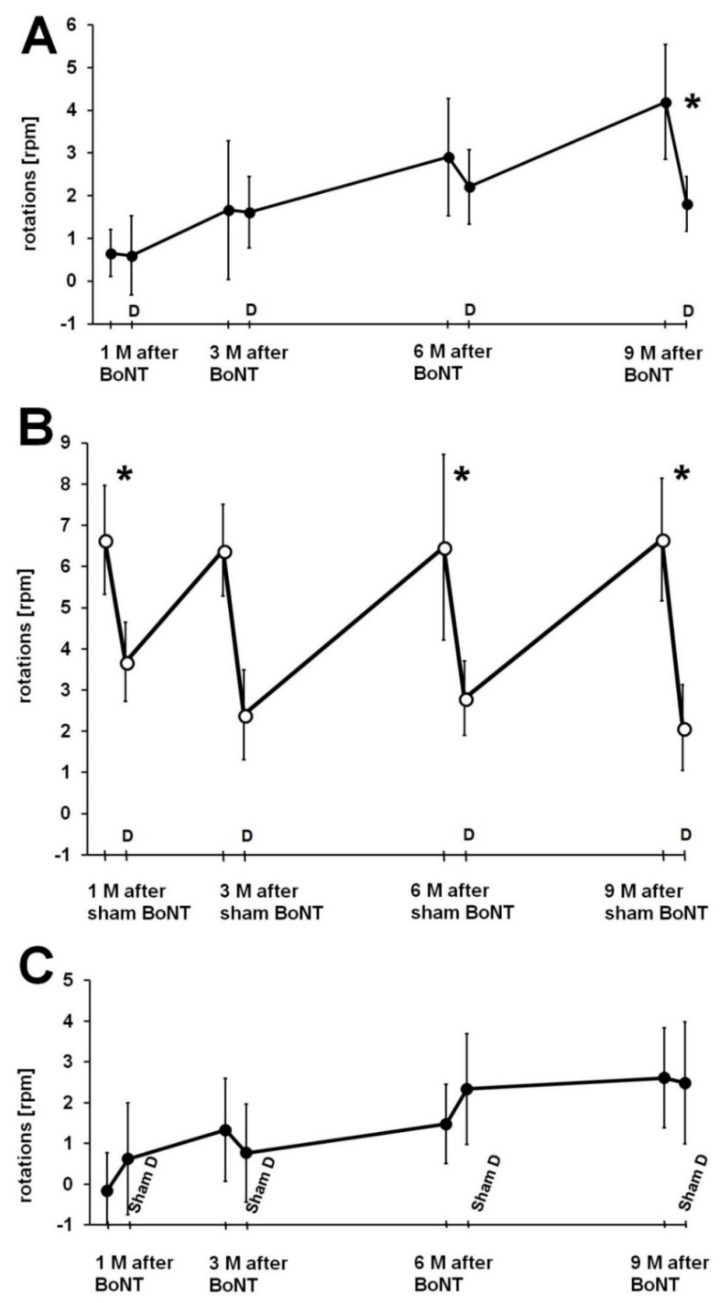
(**A**,**B**) Hemi-PD rats treated with intrastriatal BoNT-A (black dots) or vehicle (light dots) underwent repetitively apomorphine-induced rotation tests, before and 1 to 9 months after BoNT-A (**A**) or sham BoNT-A (**B**). In the second apomorphine-induced rotation test, under the influence of donepezil injected 1 h ahead, rotation rates were unchanged in the BoNT-A group (**A**) but were significantly increased in the sham BoNT-A group up to 9 months (**B**). Sham donepezil injections 1 h prior to the second apomorphine-induced rotation tests in BoNT-A-treated hemi-PD rats did not influence their rotational behavior (**C**). Asterisks indicate significant differences (* *p* < 0.05). Data are represented as mean ± SEM.

Three experimental groups of hemi-PD rats were studied (Figure 9): The first group (*n* = 6) was BoNT-A-treated 1 month after the 6-OHDA and received donepezil 1 h prior to the second rotation tests at every monitoring point (Figure 10A). The second group (*n* = 6) was sham BoNT-A-treated 1 month after 6-OHDA and received donepezil 1 h prior to the second rotation tests (Figure 10B). The third group (*n* = 6) was BoNT-A-treated 1 month after induction of hemi-PD and received sham donepezil (0.9% NaCl) 1 h prior to the second rotation tests at every monitoring point (Figure 10C). One h after injection of 2 mg kg^−1^ BW donepezil, sham BoNT-A-treated hemi-PD rats showed a significant and considerable decrease of the apomorphine-induced rotation rate of about 3 to 4 rpm (rotations in anti-clockwise direction), compared to values measured 72 h before (Figure 10B). One h after donepezil injection BoNT-A-treated hemi-PD rats only tentatively showed a decline of the turning rate, these changes being mostly insignificant (Figure 10A). Only 9 months after BoNT-A treatment, additional donepezil affected rotation behavior significantly, when the BoNT-A effect had nearly vanished (Figure 10A). Regarding BoNT-A-treated hemi-PD rats, which were injected with sham donepezil 1 h prior, a second rotation test at neither monitoring point showed significant changes in the turning rates (Figure 10C).

## 3. Discussion

Injection of 1 ng BoNT-A into the striatum of hemi-PD rats abolishes apomorphine-induced rotations for at least 3 months [12,13,14,15,16,17]. Thereafter, the apomorphine-induced rotation rate reverses [12,13,14,38]. Analogous to clinical practice in the use of BoNT-A, we evaluated the effect of repetitive BoNT-A injections (1 and 7 months after 6-OHDA) in the hemi-PD rat model using a battery of tests, including apomorphine-induced rotations and spontaneous behavior tests.

In the striatum, BoNT-A is thought to act in two main directions: ACh release of the tonically active cholinergic interneurons is blocked [1,4,5]; and the concentrations of the dopamine D_2_ receptor is reduced as shown in hemi-PD rats with D_2_ receptor upregulation, as in BoNT-A-injected normal rats [38,39]. 

### 3.1. Repetitive Intrastriatal BoNT-A Injection in Hemi-PD Rats 

#### 3.1.1. Body Weight 

Neither first nor second intrastriatal BoNT-A injections significantly influenced the body weight of the rats. Even 12 months after the second BoNT-A application, body weights of BoNT-A- (551 ± 11 g; mean ± SEM) or sham-injected rats (581 ± 14 g) did not differ significantly. Thus, repetitive intrastriatal BoNT-A had no negative effect on the health of the rats, being in line with unaltered measures after the single BoNT-A application [12,39].

#### 3.1.2. Apomorphine-Induced Rotation Test

It was shown that intrastriatal injection of 1 ng BoNT-A in hemi-PD rats ameliorated apomorphine-induced rotations for at least 3 months, and during 12 months after BoNT-A treatment, hemi-PD rats showed a gradual recurrence of the apomorphine-induced rotation rate [12,13,14,15,16,17]. We demonstrated that repetitive intrastriatal injections of BoNT-A in hemi-PD rats were well tolerated, and the second injection had an effect significantly exceeding the behavioral outcome of the first injection. The second BoNT-A injection led to a more distinct reduction of the apomorphine-induced rotation rate after 1 month: Rotation rate 1 month after first BoNT-A was 2.27 ± 0.86 rpm, and 0.17 ± 0.58 rpm (mean ± SEM) after second BoNT-A. The time dependent slope of apomorphine-induced rotations was flatter after the second BoNT-A injection: Rotation rate 3 months after first BoNT-A was 4.44 ± 0.86 rpm, and 1.32 ± 1.06 rpm after second BoNT-A. Rotation rate 6 months after first BoNT-A was 6.85 ± 1.05 rpm, and 2.20 ± 0.91 rpm after second BoNT-A. The 3 months effect of the first BoNT-A injection equaled that of the 12 months effect of the second BoNT-A application (5.08 ± 0.54 rpm). These results suggested that although 6 months after the first BoNT-A injection, the rotation rate retoured to the pre-BoNT-A value (6 months after first BoNT-A = 6.85 ± 1.05 rpm, pre-BoNT-A = 5.75 ± 1.25 rpm), a residual impact of the first BoNT-A application seemingly persisted. 

Our results concerning the more profound effect of the second intrastriatal BoNT-A injection, were in line with the prolongation of the therapeutic intervals observed following intramuscular BoNT-A injections. Rogozhin et al. [40] demonstrated in the mouse epitrochleoanconeus muscle that after repeated BoNT-A administrations, the functional recovery of neuromuscular transmission occurred slower than after a single BoNT-A injection. After a single BoNT-A injection, quantal ACh release from motor nerve terminals reached 50% of control about 6 weeks after injection; however, after repetitive BoNT-A this level was reached about 11 to 15 weeks after the last injection. Accordingly, striking structural abnormalities of neuromuscular junctions and intramuscular nerves were more pronounced after repetitive injections. Moreover, in human therapeutic BoNT-A application, more distinct and longer lasting effects, resulting in an increase in the mean duration of efficacy with the number of injections are well known [41,42,43,44,45].

We suppose that 6 months after the first intrastriatal BoNT-A injection, morphological and/or physiological parameters had not fully recovered, so that the second BoNT-A application caused a more distinct and cumulative effect, seen in the longer and more pronounced reduction of the apomorphine-induced turning rate. This phenomenon is seemingly a specific BoNT-A effect, as the apomorphine-induced rotation rate was not dependent on the time after 6-OHDA lesion, and even not influenced by sham injection experiments [38].

#### 3.1.3. Spontaneous Motor Tests

Unilateral right side 6-OHDA lesion induced marked and long-lasting stepping deficits with the left forepaw (contralateral to lesion), in both forehand and backhand directions. Neither the first nor the second ipsilateral intrastriatal BoNT-A injection changed the impairments seen in hemi-PD rats. The same was true for sensorimotor integration behavior. In the corridor task, right side hemi-PD resulted in a massive neglect of the left corridor side. The first, as well as the second ipsilateral intrastriatal BoNT-A injection, did not improve contralateral sensorimotor integration significantly. 

The outcomes of both behavioral tests reflected the motor initiation deficits of the forelimb, depending on contralateral striatal DA depletion [46,47,48,49,50,51]. DA deprivation of the striatum leads to increased GABAergic MSN projection to the EGP, which results in a disinhibition of the spontaneously active STh. As a result, a more intensely firing IGP inhibits VL. Finally, the inhibited VL neurons do not adequately activate the premotor cortex, which in turn reduces initiation of movements of the contralateral body via its crossed motor efferents [52,53,54,55]. Intrastriatal ipsilateral BoNT-A injection of 1 ng BoNT-A did not change the impairments seen in hemi-PD rats. Probably, the BoNT-A-induced changes in the DA deprived CPu concerning extracellular ACh content [56,57,58], and receptors of DA and others transmitters [59,60,61] were not adequate to improve spontaneous motor behavior. The degree of bradykinesia is associated with the degree of depletion of DA neurons [62,63]. Only animals with more than 80% striatal DA depletion exhibited similar stepping deficits [62,64,65,66], whilst animals with less depletion did not [33,67]. As in hemi-PD rats, the DA deprivation was almost complete [68,69,70,71], and consequently, not influenced by BoNT-A applications. It is not surprising that stepping behavior and sensorimotor integration were changed, neither by the first nor the second ipsilateral intrastriatal BoNT-A application.

### 3.2. Influence of Application of 2 mg kg^−1^ BW Donepezil Prior to Apomorphine-Induced Rotation Test

Hemi-PD rats treated intrastriatally with BoNT-A and injected with 2 mg kg^−1^ BW donepezil 24 h prior an apomorphine-induced rotation test, showed a significant increase of their turning rate, compared to a rotation test 72 h before. This seemingly proved our working hypothesis that the BoNT-A-induced reduction of apomorphine-induced rotation rate of hemi-PD rats was due to a reduction of the striatal ACh content. However, in sham BoNT-A-treated hemi-PD rats, we found comparable results. Moreover, time matched sham donepezil-injected BoNT-A-treated hemi-PD rats showed a significantly increase in turning rate, respectively. Therefore, we concluded that the similar increases of the rotation rate were not due to specific donepezil-induced increase of extracellular ACh content in the striatum. According to the microdialysis, studying ACh in the cortex and hippocampus of freely-moving rats following i.p. injection of 4 µmol kg^−1^ donepezil, extracellular ACh concentrations steeply increased, and showed maximal values 1 to 2 h after injection and reached baseline levels again after about 4 to 5 h [72,73,74]. Thus, apomorphine injections at an interval of 72 h were seemingly unaffected by donepezil, but could tentatively have caused a behavioral sensitization, discussed in repetitive apomorphine applications [75,76].

Remarkably, an injection of 2 mg kg^−1^ BW donepezil 1 h prior to an apomorphine-induced rotation test, in contrast to a donepezil injection 24 h prior to a rotation test, leads to a significant reduction of the turning rate in sham BoNT-A-treated rats, and to a tendential reduction of the turning rate in BoNT-A-treated rats. Obviously, the effect of donepezil in rats is limited to some hours after its systemic application, because we registered strong effects on the apomorphine-induced rotation behavior 1 h after injection, but we saw no specific alterations 24 h after application. So our results remain in line with those reports postulating a t_1/2_ of donepezil of few hours [26,27,28,29], and shown by others [72,73,74,77]. Contradicting our working hypothesis, the application of donepezil did not lead to a jump of the turning rate in BoNT-A-treated hemi-PD rats. We suppose that this is due to the systemic action of donepezil. Babiloni et al. (2014) investigated the effect of donepezil on EEG markers and motor activity in mice during short post-administration periods up to 3 h. Donepezil normalized motor activity, compared to vehicle-induced increased motor activity [78]. Comparable results were seen in a traumatic brain injury model, where Shaw et al. (2013) subsequent to daily donepezil injection (19 days) did not find motor improvement, but stated significantly impaired beam-balance time and beam-walk time at the dosage used in our experiments [79]. 

Although donepezil was shown as beneficial with respect to cognition in mild to moderate dementia [80,81,82,83], and also moderate dementia with Parkinson’s disease, donepezil therapy did not change motor PD symptoms [84,85]. Furthermore, donepezil treated dementia with Lewy body, without relevant worsening of extrapyramidal symptoms [86]. However, others stated that donepezil treatment could cause adverse motor effects in a subset of patients with parkinsonian dementia [87,88,89,90]; worsened activities of daily living mobility in patients with progressive supranuclear palsy [91]; and deteriorated motor behavior in patients with dystonic reactions [92]. 

It can be speculated that the reductions of apomorphine-induced rotations, following 1 h after donepezil, were not caused by an increase of extracellular ACh in the striatum, but by a general depression of the rats’ motor activity. The donepezil-related reduction in motor activity was measured in the sham-treated hemi-PD rats, as a reduction in apomorphine-induced rotations (Figure 10B). Seemingly, no significant donepezil-induced (Figure 10A) or sham donepezil-induced (Figure 10C) reductions in motor activity were measured in the BoNT-A-treated hemi-PD rats, as those rats performed fewer rotations per se. 

We conclude, that the systemic administration of donepezil, irrespective of the time prior to an apomorphine-induced rotation test, was not a qualified method to prove the ACh dependence of the effect of intrastriatal injection BoNT-A. Application of donepezil 24 h ahead of apomorphine had no specific effect, as the temporal increase of extracellular ACh in the brain was already abrogated. Application of donepezil 1 h before apomorphine likely did not have the hypothetically expected effect, i.e., reversing the BoNT-A-induced reduction of apomorphine-induced rotation behavior, caused by the increase of extracellular ACh, as the general motor-depressive effect of donepezil exceeded that of the increase of extracellular ACh in the striatum.

## 4. Conclusions 

Firstly, data showed that repeated intrastriatal BoNT-A injections in the hemi-PD rat model were possible and well tolerated. In hemi-PD rats, the second intrastriatal BoNT-A injection had a more intense and longer lasting effect on the reduction of apomorphine-induced rotations, but like the first one, it did not affect forelimb akinesia and lateralized sensorimotor integration. Secondly, systemic donepezil injection prior to testing the apomorphine-induced rotation behavior is not qualified to prove the dependency of striatal extracellular ACh content for turning rate reductions after BoNT-A treatment.

These different effects of BoNT-A application suggest that intrastriatally applied BoNT-A acts both as an inhibitor of ACh release and influences transmitter receptors, especially D_2_ receptor expression, and thereby, affects the basal ganglia circuitries. Therefore, the evaluation of receptor densities of all important striatal transmitters is the subject of ongoing studies of our group.

## 5. Materials and Methods

### 5.1. Animals

All experiments were started with 2.5 months old, male Wistar rats (strain Crl:WI BR, Charles River Wiga, Sulzfeld, Germany) weighing 295–305 g. Animals were housed in standard cages at 22 °C ± 2 °C under a 12 h light/dark cycle, with free access to water and food. All procedures used in the present study complied with the guidelines on animal care. The experiments were approved by the local Animal Research Committee of the state of Mecklenburg-Western Pomerania (LALLF M-V 7221.3-1.1-003/13 from 26 April 2013.

### 5.2. Induction of Hemiparkinsonism

All surgeries were carried out under aseptic conditions and deep anesthesia (50 mg kg^−1^ BW) ketamine and 4 mg kg^−1^ BW xylazine). To induce an experimental hemi-PD syndrome, a unilateral injection of 24 µg 6-OHDA dissolved in 4 µL 0.1 M citrate buffer was performed into the right medial forebrain bundle (MFB), using a stereotactic frame (Stoelting, Wood Dale, IL, USA). The injection coordinates with reference to bregma were: AP = −2.3, L = 1.5, V = −9.0, as described in Reference [93]. The success of the lesion was verified by measurement of the apomorphine-induced rotation rate [94,95], 4 weeks after 6-OHDA injection. Rats (*n* = 45) that displayed an apomorphine-induced rotation rate of at least 4 rotations per minute to the left side, were successfully lesioned, indicating unilateral death of about 97% of the nigrostriatal dopaminergic neurons [95]. Only those rats were used for further experiments. 

### 5.3. Body Weight

Body weights were measured at the following time points: 6-OHDA injection, first and second BoNT-A or vehicle injections, as well as at every monitoring point and 12 months after the second BoNT-A or vehicle applications.

### 5.4. Injection of BoNT-A into the Striatum

About 6 weeks after the 6-OHDA injection, the animals underwent a next stereotactic surgery (Figure 2). Either a solution of BoNT-A dissolved in phosphate-buffered saline supplemented with 0.1% bovine serum (prepared from BoNT-A powder, Lot No. 13028A1A; List, Campbell, CA, USA; purchased via Quadratech, Surrey, UK) or vehicle (sham BoNT-A) solution was injected into the right CPu at two sites [12,14,15]. Hemi-PD rats were treated with either 1 ng BoNT-A (*n* = 29) or vehicle (*n* = 16). The respective coordinates with reference to bregma were: AP = +1.3/−0.4 mm, L = 2.6/3.6 mm to the right, and V = −5.5 mm. Animals received a 2 × 1 µL BoNT-A solution containing a total of 1 ng BoNT-A or vehicle solution. Animals for the repetitive BoNT-A injection experiment underwent a second BoNT-A or a sham injection with vehicle solution, 6 months after the first one, respectively (Figure 2).

### 5.5. Apomorphine-Induced Rotation Test

The rate of apomorphine-induced rotations, served as a measure for the extent of the basal ganglia circuit disturbance by the unilateral lesion of the SNpc [94,95].

The apomorphine-induced turning rate was ascertained 4 weeks after the 6-OHDA lesion, and 1, 3, and 6 months after the first BoNT-A injection, as well as 1, 3, 6, 9, and 12 months after the second BoNT-A treatment. Two experimental groups were studied: hemi-PD rats treated with BoNT-A (*n* = 16), and hemi-PD rats injected with vehicle solution (*n* = 8) (Figure 2). 

Apomorphine was injected s. c. (0.25 mg kg^−1^) and the animals’ turns registered on a self-constructed automated rotometer device over 40 min. In right-sided hemi-PD rats, the apomorphine-induced complete anti-clockwise 360° turns were expressed as positive values (Figure 4, Figure 8 and Figure 10).

### 5.6. Spontaneous Motor Tests

Stepping test and corridor task were performed before 6-OHDA lesion, 4 weeks thereafter, and 1, 3, and 6 months after the first ipsilateral intrastriatal injection of BoNT-A or sham BoNT-A; and 1, 3, 6, 9, and 12 months after the second BoNT-A or vehicle injection (Figure 2). 

#### 5.6.1. Adjusting Steps

Before evaluating adjusting steps, rats were handled by the experimenter during 3 days to adapt to the test procedure [33,96,97]. Tests were performed twice per day on 3 successive days. Briefly, the rat was held with one hand softly blocking both its hind limbs and the restrained forelimb, with the unrestrained forepaw touching the table. The rat was moved slowly sideways across the table (90 cm in 5 s) and the number of adjusting steps of the unrestrained left or right forelimb was counted, whilst moving in the forehand and backhand directions. Subsequently, the forehand and backhand steps of left and right paws were evaluated using the video recorded sessions, which allowed counting of the number of adjusting steps by an investigator blinded to the state of the rats. 

#### 5.6.2. Corridor Task 

Lateralized response selection was examined using the version, according to Grealish et al. [98]. Prior to testing rats were food restricted for 3 days, and maintained at about 90% of free-feeding BW during habituation and testing, as described in Reference [99]. Rats were adapted to the self-constructed alleyway (240 cm long × 7 cm wide × 23 cm deep) for 10 min each on 2 successive days, with some scattered sugar pellets (Ain-76A Rodent Tablet 20 mg TestDiet, Richmond, IN, USA) along the floor of the corridor. Each day, the animals started from different ends of the corridor. For final testing, on day 1, rats were first placed in an identical but empty corridor for 5 min for adaptation, and then to the end of the testing corridor. In the testing corridor, bowls (2 cm in diameter, distance between the bowls 15 cm) were placed on the left and right sides, containing 5 pellets each. Rats were allowed for 5 min to retrieve pellets from either side of their body, as detailed in References [50,100]. The retrievals of the right side (ipsilateral) and left side (contralateral) were counted, and the data expressed as the percentage of left- or right-side retrievals on the total number of retrievals. The side is defined according to the rat’s body axis. A “retrieval” involved a nose poke into a bowl, whether or not pellets were taken from it, as outlined in References [98,101,102].

### 5.7. Donepezil Modifying Apomorphine-Induced Rotations

In different experiments the possible effect of donepezil on apomorphine-induced rotation rates was evaluated. Hemi-PD rats intrastriatally injected with BoNT-A (1 ng) or sham BoNT-A were twice tested for apomorphine-induced rotations, at an interval of 72 h. At 24 h or 1 h prior to the second test for apomorphine-induced rotations, donepezil (2 mg kg^−1^ BW) or sham donepezil (0.9% NaCl) was injected i.p. (Figure 8 and Figure 10).

Thus, a total of 7 groups were tested: (i) hemi-PD + BoNT-A + 24 h donepezil (*n* = 15); (ii) hemi-PD + sham BoNT-A + 24 h donepezil (*n* = 9); (iii) hemi-PD + BoNT-A + 24 h sham donepezil (*n* = 14); (iv) hemi-PD + sham BoNT-A + 24 h sham donepezil (*n* = 7); (v) hemi-PD + BoNT-A + 1 h donepezil (*n* = 6); (vi) hemi-PD + sham BoNT-A + 1 h donepezil (*n* = 6); and (vii) hemi-PD + BoNT-A + 1 h sham donepezil (*n* = 6)—(not done: hemi-PD + sham BoNT-A + 1 h sham donepezil). Groups i–iv were tested 1 to 4 months after BoNT-A or sham BoNT-A, and groups v–vii 1 to 12 months after BoNT-A or sham BoNT-A.

### 5.8. Data Analysis

The results were presented as means ± SEM. Computations and statistics of donepezil modifying apomorphine-induced rotations were performed with Excel^®^. For comparison of rotation rates and slopes of rotation rates between two animal groups at the same monitoring point, the unpaired Student’s *t*-Test was performed. For investigation of rotation rates and slopes of the rotation rate of one animal group at different monitoring points, the paired Student’s *t*-Test was performed. In all cases, *p* values ≤ 0.05 were considered significant and *p* values < 0.01 were considered highly significant.

Data of body weights and spontaneous motor tests, i.e., stepping test and corridor task, were subjected to two-way ANOVA using SigmaPlot 11 Software (Systat Software, Inc., San Jose, CA 95110, USA). The Holm-Sidak approach was used for adjustment for multiple testing, for post hoc comparisons. A critical value for significance of *p* ≤ 0.05 was used. 

## Abbreviations

6-OHDA	6-hydroxydopamine
ACh	acetylcholine
AChE	acetylcholinesterase
BoNT-A	botulinum neurotoxin-A
BW	body weight
CPu	caudate-putamen (striatum)
DA	dopamine
D_2_	dopamine D_2_ receptor
EGP (=LGP)	external (lateral) globus pallidus
GABA	gamma-aminobutyric acid
hemi-PD	hemiparkinsonian
IGP (=MGP)	internal (medial) globus pallidus
M	slope/drawdown
MFB	medial forebrain bundle
MSN	medium spiny neuron
PD	Parkinson’s disease
PPN	pedunculopontine nucleus
RPM/d	(revolutions per minute)/day
SNAP-25	synaptosomal-associated protein-25
SNARE	*N*-ethylmaleimide-sensitive-factor attachment receptor
SNpc	substantia nigra pars compacta
STh	subthalamic nucleus
VL	ventrolateral thalamic nucleus
